# Newcastle disease virus expressing an angiogenic inhibitor exerts an enhanced therapeutic efficacy in colon cancer model

**DOI:** 10.1371/journal.pone.0264896

**Published:** 2022-04-05

**Authors:** Fanrui Meng, Yukai Cao, Han Su, Tianyan Liu, Limin Tian, Yu Zhang, Jiarui Yang, Wei Xiao, Deshan Li

**Affiliations:** 1 School of Life Science, Northeast Agricultural University, Harbin, China; 2 State Key Laboratory of New-Tech for Chinese Medicine Pharmaceutical Process, Jiangsu Kanion Pharmaceutical Co., Ltd., Lianyungang, China; UTHSC: The University of Texas Health Science Center at Houston, UNITED STATES

## Abstract

Newcastle disease virus (NDV)-mediated gene therapy is a promising new approach for treatment of cancer but shows limited anti-angiogenesis. VEGF-Trap plays a vital role in anti-angiogenesis. To enhance the anti-tumor effect of NDV, VEGF-Trap gene was incorporated into the genome of rNDV in this study (named rNDV-VEGF-Trap). Results showed that rNDV-VEGF-Trap reduced cell growth ratio by 85.37% and migration ratio by 87.9% in EA.hy926 cells. *In vivo* studies, rNDV-VEGF-Trap reduced tumor volume and weight of CT26-bearing mice by more than 3 folds. Immunohistochemistry analysis of CD34 showed rNDV-VEGF-Trap significantly decreased the number of vascular endothelial cells in the tumor tissues. Moreover, Western blot analysis demonstrated that treatment with rNDV-VEGF-Trap significantly decreased the phosphorylation levels of AKT, ERK1/2 and STAT3 and increased the expression levels of P53, BAX and cleaved caspase-3 in the tumor tissue. In addition, to evaluate the toxicity of rNDV-VEGF-Trap, serum chemistries were analyzed. The results showed that rNDV-VEGF-Trap caused insignificant changes of creatinine levels, alanine aminotransferase and aspartate transaminase. Furthermore, administration of rNDV-VEGF-Trap did not cause the diarrhoea, decreased appetite, weight decrease and haemorrhage of the experimental mice. These data suggest that rNDV-VEGF-Trap exhibits an enhanced inhibition of CT26-bearing mice by enhancing anti-angiogenesis and apoptosis and may be a potential candidate for carcinoma therapy especially for colon cancer.

## Introduction

Colon cancer is one of the main causes of cancer death in humans [1]. It is similar to most other malignancies possessing the risk of malignant invasion and metastasis [2, 3]. One-half of patients with colon cancer will develop liver or lung metastasis, which are associated with abundant vascular distribution [4]. Currently, surgery, radiotherapy and chemotherapy are the major strategies for colon cancer. However, the high recurrence rate and adverse reactions decreased overall survival rate [5]. Therefore, optimizing the treatment strategy is a major challenge to improve the survival rate [6–8]. Besides traditional methods, immunotherapy has become a hot field, which includes oncolytic viruses, tumor vaccines and immune checkpoint inhibitors etc. [9–14]. With the development of genetic engineering and related disciplines, oncolytic viruses have been widely applied to treat colon cancer in recent years [15].

Newcastle disease virus (NDV) is affiliated to the family Paramyxoviridae, which is an avian paramyxovirus type I virus [16, 17]. The 15186 nucleotides of NDV genome encode six structural proteins, which are nucleocapsid protein (NP), phosphoprotein (P), RNA-dependent RNA polymerase (L), matrix protein (M), hemagglutinin-neuraminidase (HN) and fusion protein (F) [18, 19]. NDV causes severe infection in avian species, but there is no significant symptoms to humans. NDV is safe in humans due to host range restriction and there is no pre-existing antibody to NDV in the human population. NDV is antigenically distinct from common human pathogens [20]. Moreover, it exhibits oncolytic ability through specifically replicates in tumor cells rather than normal cells [21, 22]. The oncolytic potency relies on the ability to induce apoptosis in infected cancer cells [23, 24]. However, the clinical application of NDV isn’t satisfactory. The reason may be that anti-angiogenesis effect of NDV is still limited [25, 26].

Angiogenesis plays a crucial role in tumor growth and metastasis [27, 28]. The growth, local tissue invasion and distant metastasis of colon cancer are highly dependent on angiogenesis [29, 30]. Colon cancer is one of the ideal angiogenesis-dependent solid tumors. In tumor cells, the activation of oncogenes and inactivation of tumor suppressor genes lead to the upregulation of VEGF [31, 32]. Overproduction of VEGF leads to abnormal changes in tumor vessels, such as distortion, fragmentation, and lack of pericytes. These dysfunctional and leaky vessels promote the development of tumor tissue microenvironment, which limits the delivery of anti-tumor drugs to the targets [33]. VEGF and its signaling pathway represent attractive targets for the treatment of cancer, because the growth factor plays an important pro-angiogenic cytokine role in various cancer types [34]. A previous study describes anti-angiogenic therapy using vascular endothelial growth factor trap (named VEGF-Trap or aflibercept), which is an effective protein that blocks the tumor-associated VEGF signaling pathway. VEGF-Trap, as a fusion protein, is composed of some extracellular domains of VEGF 1 receptor and VEGF 2 receptor and FC segment of human IgG antibody [35]. VEGF-Trap blocks the signal transduction of VEGF/VEGFR and inhibits angiogenesis and the tumor growth by binding with VEGF [36]. However, repeated injections of VEGF-Trap lead to adverse reactions, such as aspartate transaminase (AST) increase, serum creatinine increase, alanine aminotransferase (ALT) increase, diarrhea, decreased appetite, weight decrease and haemorrhage of the patients [37]. Therefore, the treatment of VEGF-Trap still needs to overcome these limitations.

In this study, we attempt to enhance the antitumor effect of NDV by modifying it to express VEGF-Trap. Antitumor efficacy and security were evaluated *in vitro* and *in vivo*. Our results suggest that the virus possesses a significant inhibitory effect on vascular endothelial cells line EA.hy926 and shows an enhanced oncolytic activity and security in the CT26-bearing model mice.

## Materials and methods

### Virus and cell lines

The strain *E*.*coli* DH5α, strain *E*.*coli* STBL2, recombinant Newcastle disease virus vector, VEGF-Trap gene, auxiliary plasmid (PTM-NP, PTM-P and PTM-L), human colon cancer cell line (HCT116), human umbilical vein endothelial cell line (EA.hy926), mouse colon cancer cell line (CT26), mouse breast cancer cell line (4T-1), hamster kidney cell line (BHK-21), were supplied by northeast agricultural university biological pharmaceutical teaching and research section. CT26 and 4T-1 were maintained in RPMI 1640 supplemented with 10% (v/v) FBS, 1% (v/v) penicillin/streptomycin. BHK-21 were maintained in DMEM containing 10% new-born calf serum (NCS) and 1% penicillin/streptomycin. HCT116 was maintained in McCoy’5A supplemented with 10% (v/v) FBS, 1% (v/v) penicillin/streptomycin. All cell lines were authenticated using Short Tandem Repeat profiling, tested for mycoplasma contamination and grown at 37°C humidified incubator with 5% CO_2_ atmosphere.

### Other reagents

DMEM, McCoy’5A, RPMI 1640 were purchased from GIBCO company, and fetal bovine serum was purchased from PAN-Biotech; Restriction enzymes (*Pme*Ⅰ, *Sac*Ⅱ), rTaq DNA polymerase, T4 DNA ligase purchase since NEB company; DNA Marker, PMD18-T and dNTPs were purchased from TaKaRa Company. Lipofectmine 3000 transfection reagent purchased from Invitrogen company; The plasmid extraction kit was purchased from OMEGA; DNA recovery kit and PCR product purification kit were purchased from Qiagen Company. DNA PCR primers were synthesized by Shanghai Sangon. Anti-human IgG4 Fc (HRP) antibodies, anti-mouse Bcl-2, VEGF-Trap and CD34 antibodies were purchased from Abcam. Anti-mouse β-actin, caspase-3, BAX, STAT3, AKT, p44/42MAPK(ERK1/2), phospho-STAT3 (named P-STAT3), phospho-AKT (named P-AKT) and phospho-p44/42MAPK(ERK1/2) (named P-ERK1/2) antibodies were purchased from cell signaling technology.

### Recombinant Newcastle disease virus

The clone30 strain was used to provide a backbone for construction of the recombinant virus, which could cause syncytia formation in mammalian cells. Reverse-genetics technology was used for the construction of rNDV [38]. In order to improve the oncolytic effect of the virus, the recombinant plasmid was constructed by exchanging the F gene of lentogenic strain Clone30 with Anhinga, and then the chimeric virus rNDV was constructed [24] and sequenced by reverse transcription PCR for fidelity.

### Analysis of viral growth in CT26 cell line

Viral growth was determined in the CT26 cell line. Cells planted in 24-well plates were infected with 0.1 MOI rNDV or rNDV-VEGF-Trap. The supernatants were collected at 12, 24, 36, 48, 60 and 72h post infection. The viral concentration was measured at end-point titration on CT26 cells and calculated as 50% tissue culture infective dose (lgTCID_50_) per mL.

### Determination of exogenous VEGF-Trap protein expression

CT26 cells (5 × 10^6^ cells) were respectively infected with 1 MOI rNDV-VEGF-Trap and rNDV and the control group received PBS. The methods were accorded to description of Ying An’s paper [24]. After 24h incubation, cell supernatant and cells resuspended in lysis buffer supplemented with proteases inhibitor were harvested for Western blot. Samples were separated by 10% sodium dodecylsulfate-poly acrylamide gel electrophoresis (SDS-PAGE), and transferred to a nitrocellulose membrane. The blot was visualized by chemiluminescence and autoradiography using X-ray film. Each gel was tested with mouse anti-human IgG4 Fc (HRP) antibody to identify VEGF-Trap.

### Cell viability assay

Cell viability was quantified by a short-term microculture tetrazolium (MTT) assay [24]. Approximately 1–2 × 10^4^ CT26, 4T-1, HCT116 and EA.hy926 cells were plated into 96-well plates in complete medium. Then, these cells were infected with rNDV-VEGF-Trap, rNDV at the dose of 0.01 MOI, 0.1 MOI, 1 MOI, and 10 MOI in triplicate. 20 μL MTT solutions (5 mg/mL in sterile phosphate-buffered saline) were added to the cell after 48h of incubation. After 4 hours, the MTT solution in the wells was discarded, then 150 μL dimethyl sulfoxide (DMSO) was added. The absorption was measured by a microplate reader at 490 nm (OD490). The cell viability was converted and expressed as the percentage of the control. The cells without any treatment were used as negative control.

Inhibition rate = (control group OD—treatment group OD)/control group OD × 100%

### Migration assay

The effect of rNDV-VEGF-Trap on cell migration was measured by wound healing experiments [24]. The same EA.hy926 cell numbers were respectively seeded into 6-well plates as control and treatment groups. Then, a cell-free area of confluent monolayer cells was created by scratching with a 1 mL pipette tip. After washing the isolated cells, monolayer cells were cultured in a maintenance medium containing 2% FBS. The cells were incubated with 1 MOI rNDV or rNDV-VEGF-Trap for 24h and 48h, then washed with PBS and photographed. The representative images were captured with a camera (Nikon, Japan) attached to a microscope. For comparing the migration rate, scratches were photographed in three separate fields, and then the scratch area was measured by ImageJ. The experiment was repeated three times.

### Analysis of tumor growth in colon cancer model

All procedures involving animals were according to the guidelines issued by National Institute of Health and the Institutional Animal Care and Use Committee of Northeast Agriculture University. Healthy 6-week-old female BALB/c mice with a weight of 19 ± 1 g were permitted to feed and drink freely. CT26 cells (1 × 10^6^) were subcutaneously implanted into the right groin of mice and tumors were allowed to grow until the average diameter reached 5–8 mm. Reference to previous publications [23, 24], Mice were randomly divided into different groups (*n* = 10), and intratumorally injected with allantoic fluid or 1 × 10^7^ pfu of the indicated viruses every two day. Tumor volumes were calculated using the following formula: tumor volume (*V*) *=* 4/3 × *π* × *S*2/2 × *L*/2, where *S* is the smallest measured diameter and *L* is the largest diameter. Animals were humanely culled when tumor size reached 18 mm in any dimension or at defined experimental time points. After the treatment, animals of each group were sacrificed, their tumors and spleens were excised, weighted, frozen, or fixed in 4% paraformaldehyde.

### Histological analysis and immunohistochemical (IHC) assay

According to the method of Elham Assareh paper [39], the tumor tissues of tumor-bearing mice were fixed with 4% formalin at room temperature for 2 days, treated with graded concentrations of ethanol and xylene, and embedded in paraffin. The sections of 4–5 mm were mounted on adhesive glass slides. Tumor tissue sections were stained with Hematoxylin and eosin (HE) for investigation of morphological changes in treatment and control groups. IHC staining analysis was performed to localize specific tissue antigens. The sections were incubated at 4°C with the primary mouse monoclonal antibodies CD34 (1:2500) for overnight. Antigens is detected with 3,3-diaminobenzidine (DAB). All images were analyzed using Image J software.

### Western blot analysis

CT26 tumor tissues were lysed with RIPA buffer containing protease and phosphatase inhibitors (Beyotime, China) as described by Maryam Farizaneh Behelgardi report [40]. After centrifugation, the protein samples were treated with 5 × loading buffer (Beyotime, China). Protein concentrations were determined by the BCA quantitative kit (Beyotime, China). Equal amounts of total proteins were added into 12% sodium dodecyl sulfate-polyacrylamide gel for electrophoresis. Proteins were transferred to polyvinyl difluoride (PVDF) membranes followed by blocking with 5% non-fat milk for 2h. The primary antibodies including anti-P-AKT, anti-total-AKT, anti-P-STAT3 (1:2000), anti-P-ERK1/2, anti-total-STAT3, anti-total-ERK1/2, anti-P53, anti-BAX, anti-Bcl-2 and anti-caspase-3 (1:1000) were incubated overnight at 4 °C. After three times washing in Tris Buffered Saline-Tween, the membranes were incubated with an appropriate HRP-conjugated secondary antibody for 1 h at room temperature. Protein bands were visualized using ECL reagent.

### Toxicity study

Based on the exposure of VEGF-Trap in clinical treatment of various adverse reactions, the diarrhoea, appetite, weight and haemorrhage of the experimental mice were recorded throughout the treatment phase. At the end of the animal experiment, whole blood was collected through the eyeballs of the experimental mice. Then the serum was isolated from whole blood for determination of serum concentrations of AST, ALT and creatinine. All key organs were observed and viral residues were detected in the normal tissues.

### Statistical analysis

The statistical significance of quantitative data between different groups were determined with GraphPad prism software (Version 5.01, GraphPad Software Inc., La Jolla, California). All data were expressed as mean ± SEM, significance was determined by performing one- or two-sided Student’s t tests and defined as a *p* value < 0.05, *p* value < 0.01.

## Results

### Generation of rNDV-VEGF-Trap

**Fig 1:**
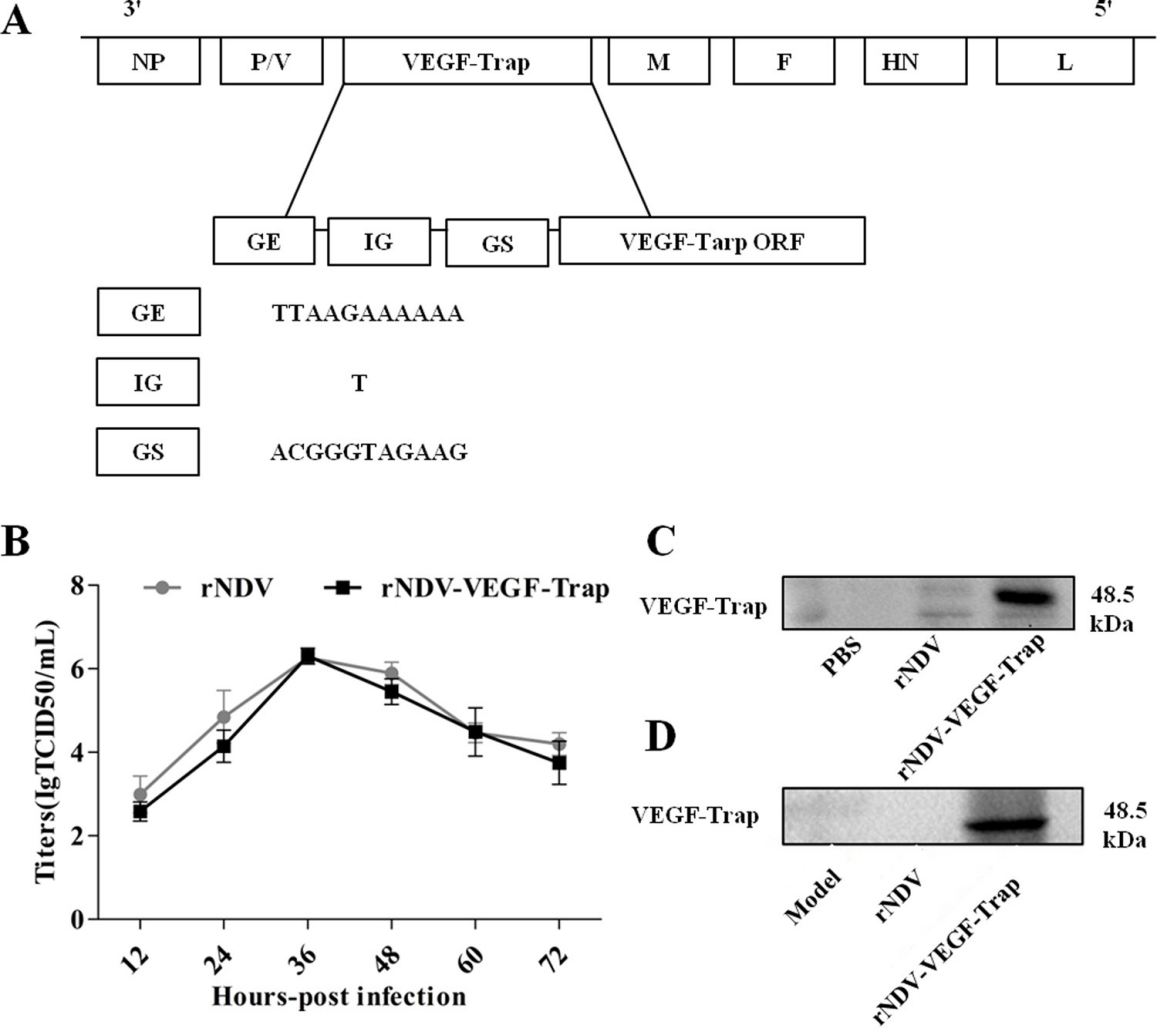
Schematic representation of the genomes, characterization and VEGF-Trap protein of rNDV-VEGF-Trap. **(A)** Diagram showed insertion of the VEGF-Trap gene into the rNDV genomes at the position between the P and M genes. Recombinant NDV were rescued by transfecting BHK-21 cells with incorporated plasmid along with the helper plasmid *PTM-NP*, *PTM-P* and *PTM-L*. The culture supernatant was harvested at 72h post transfection and inoculated into 10 days SPF embryo chicken eggs. After 3 days, the allantoic fluid was harvested and the titers were determined by hemagglutination Assay (HA) test. **(B)** Growth curves of rNDV-VEGF-Trap. CT26 cells were infected with rNDV and rNDV-VEGF-Trap at 0.1 MOI. Cell monolayers were lysed at 12, 24, 48, 60 and 72h post-infection for intracellular titer measurement by TCID50. **(C)** The expression of VEGF-Trap proteins in CT26 cells. CT26 cells were respectively infected with 1 MOI rNDV-VEGF-Trap or rNDV and the control group received PBS. After 24h incubation, cell supernatant and cells resuspended in lysis buffer supplemented with proteases inhibitor were harvested for Western blot. Result showed that the VEGF-Trap expression was detected in the mixture of cell lysate and supernatant. **(D)** *In vivo*, tumor tissues were selected from each group and assayed using Western blot. Result demonstrated that the expression of VEGF-Trap in the tumor tissues of rNDV-VEGF-Trap treated group was significantly higher than that in rNDV-treated group and model group.

The VEGF-Trap gene was incorporated at the position between the phosphoprotein and matrix protein genes of the rNDV genome (Fig 1A). Recombinant NDV were then rescued by transfecting BHK-21 cells with incorporated plasmid along with the helper plasmid *PTM-NP*, *PTM-P* and *PTM-L*. The culture supernatant was harvested at 72h post transfection and inoculated into 10 days SPF embryo chicken eggs. After 3 days, the allantoic fluid was harvested and the titers were determined by hemagglutination Assay (HA) test. The successful generation of rNDV-VEGF-Trap virus were evaluated by the high titers of virus.

### Growth and VEGF-Trap protein expression of rNDV-VEGF-Trap

In order to verify the proliferation ability of rNDV-VEGF-Trap in tumor cells, CT26 cells were infected with 0.1 MOI rNDV or rNDV-VEGF-Trap. The supernatant was harvested at different time points. Then the viral titers in the supernatants were determined in triplicate. Compared with rNDV, rNDV-VEGF-Trap has no significant difference in the kinetics of replication (Fig 1B). To investigate the expression of VEGF-Trap protein, CT26 cells were treated with 1 MOI rNDV-VEGF-Trap. After 24h incubation, cell supernatant and cells resuspended in lysis buffer supplemented with proteases inhibitor were harvested for Western blot. Result showed that the VEGF-Trap expression was detected in the mixture of cell lysate and supernatant (Fig 1C). *In vivo*, tumor tissues were selected from each group and assayed using Western blot. Result demonstrated that the expression of VEGF-Trap in the tumor tissues of rNDV-VEGF-Trap treated group was significantly higher than that in rNDV-treated group and model-treated group (Fig 1D). Overall, these results indicate that rNDV-VEGF-Trap expresses VEGF-Trap protein at a high level.

### rNDV-VEGF-Trap suppresses tumor cells and vascular endothelial cells growth

**Fig 2:**
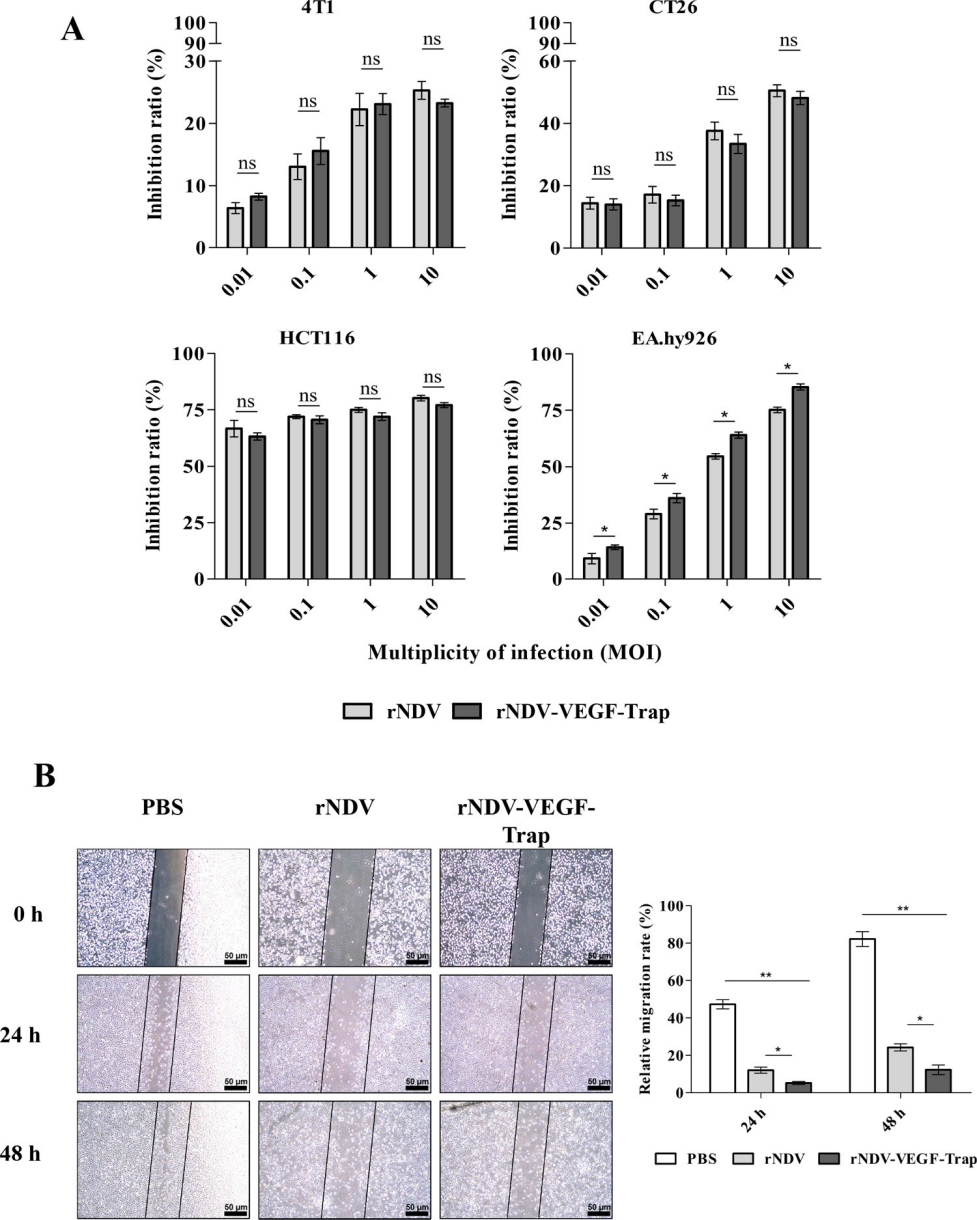
In vitro studies. **(A)** Cytotoxic effects of rNDV-VEGF-Trap on cancer cells and vascular endothelial cells. 4T-1, CT26, HCT116, and EA.hy926 cells incubated with PBS were used as controls. MTT method was used to measure the cell density. The data are the mean ± SEM of triple samples. **(B)** Inhibition of cell migration by recombinant virus. Wound healing assay was performed on EA.hy926 cells, the cells were infected with 1 MOI rNDV-VEGF-Trap or rNDV and cell migration was evaluated at regular time points post-infection. The number of migrated cells were assessed by microscope. The assay was performed in triplicate. The data are the means ± SEM of triple samples (ns means no significance; *P < 0.05; **P < 0.01; ^##^*P* < 0.01).

To better characterize the rNDV-VEGF-Trap vrus in comparison to the parental virus NDV, the direct cytotoxic activity of rNDV-VEGF-Trap was measured in CT26, 4T-1, HCT116, EA.hy926 cells in this study. Results showed that there is no significant difference in other three cell types between rNDV and rNDV-VEGF-Trap. The growth inhibition rates of rNDV and rNDV-VEGF-Trap treated group increased along with virus concentration in four cell lines (Fig 2A). These data indicate that there is no significant difference in cytotoxic activity between rNDV and rNDV-VEGF-Trap. However, when EA.hy926 were infected with rNDV-VEGF-Trap or rNDV of 10 MOI for 48h, the inhibition rates were respectively 85.37% ± 3.20% and 74.26% ± 2.12%. These data suggest that rNDV-VEGF-Trap significantly inhibits the growth of EA.hy926 cells compared with rNDV.

### Effects of rNDV-VEGF-Trap on EA.hy926 cell migration

Scratch test was used to investigate changes in the migration of EA.hy926 cells after treatment with rNDV and rNDV-VEGF-Trap for 48h. The migration rates of EA.hy926 cell in PBS group, rNDV group and rNDV-VEGF-Trap group were respectively 81.79%, 26.5% and 12.1% at 48h (Fig 2B). The results showed that rNDV-VEGF-Trap further inhibited EA.hy926 cell migration compared with rNDV.

### rNDV-VEGF-Trap further suppresses tumor growth in mice

**Fig 3:**
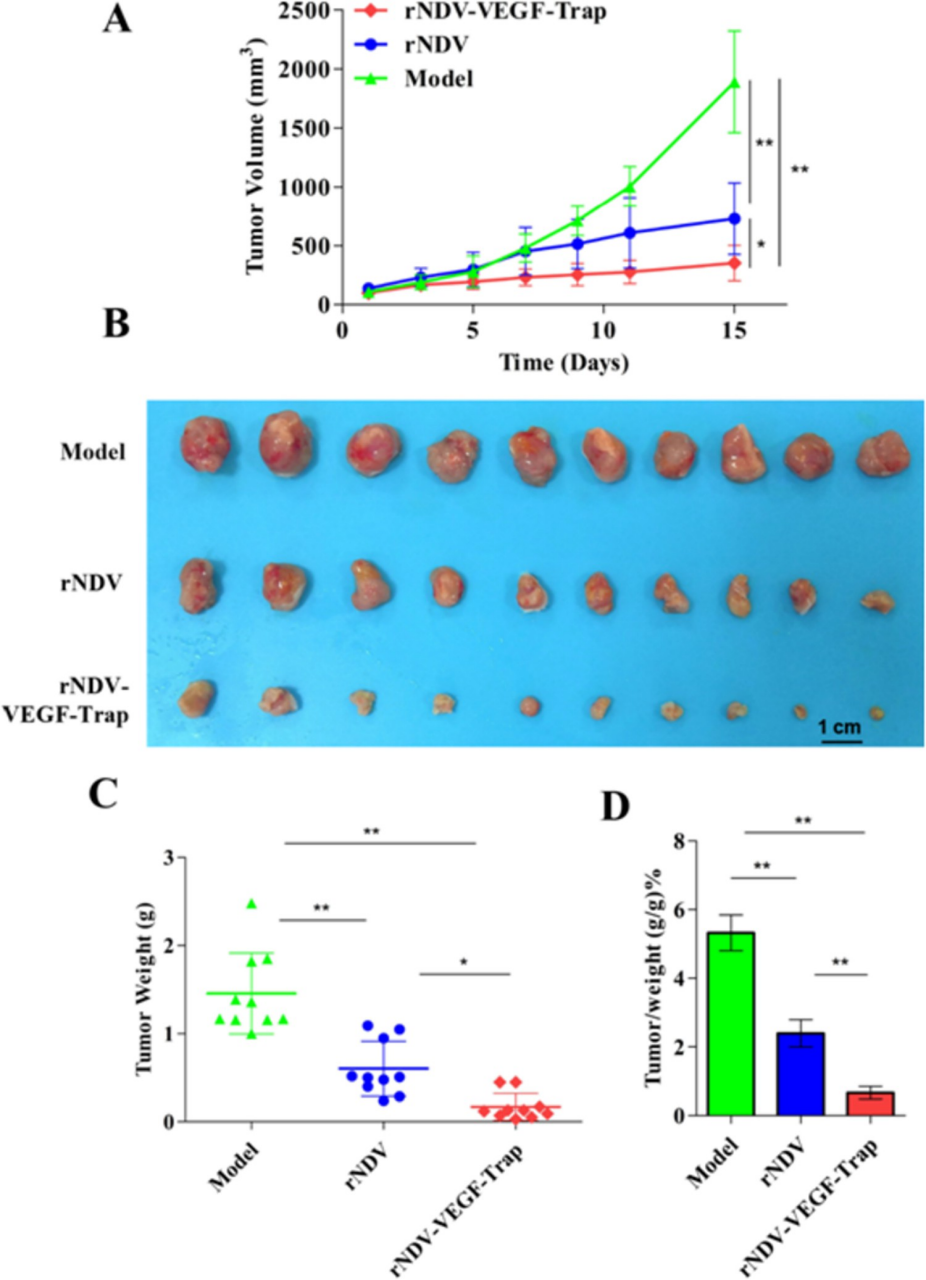
rNDV-VEGF-Trap further suppresses tumor growth of CT26-bearing mice. Six-week-old female BALB/c mice were injected with 10^6^ CT26 cells in the right groin. When the tumor size reached about 100 mm^3^, the mice were intratumorally treated with rNDV or rNDV-VEGF-Trap at 10^7^ pfu. The mice were euthanized and necropsy was performed after 15 days of treatment. **(A)** The tumor volume of CT26-bearing mice was measured in two dimensions every other day using digital calipers. **(B)** Tumor collected at the end of the experiment. **(C)** Tumor Weight. **(D)** The ratio of tumor weight to body weight. Model: mice treated with allantoic fluid (*n* = 10); ns: no significance; *P < 0.05; **P < 0.01.

When the tumor size reached about 100 mm^3^, the mice were divided into three groups. Their tumor volume and weight were recorded every 2 days. The average tumor sizes of the model group, rNDV group and rNDV-VEGF-Trap group were respectively 1889.18±408.16 mm^3^, 728.49±287.41 mm^3^ and 351.1±142.58 mm^3^ (Fig 3A). After necropsy, tumors excised from each group showed that rNDV-VEGF-Trap significantly ameliorates tumor texture and vascular density compared to both model and rNDV groups (Fig 3B). Moreover, the average tumor weights of each group were respectively 1.456±0.459 g, 0.603±0.311 g and 0.169±0.154 g (Fig 3C). The average rates of tumor weight in each group were respectively 5.34±0.81, 2.22±0.33 and 0.64±0.24 (Fig 3D). Taken together, these data demonstrate that treatment with rNDV-VEGF-Trap further inhibits tumor growth compared with rDNV or model group in the colon cancer model.

### rNDV-VEGF-Trap shows enhanced antineoplastic activity and anti-angiogenesis

**Fig 4:**
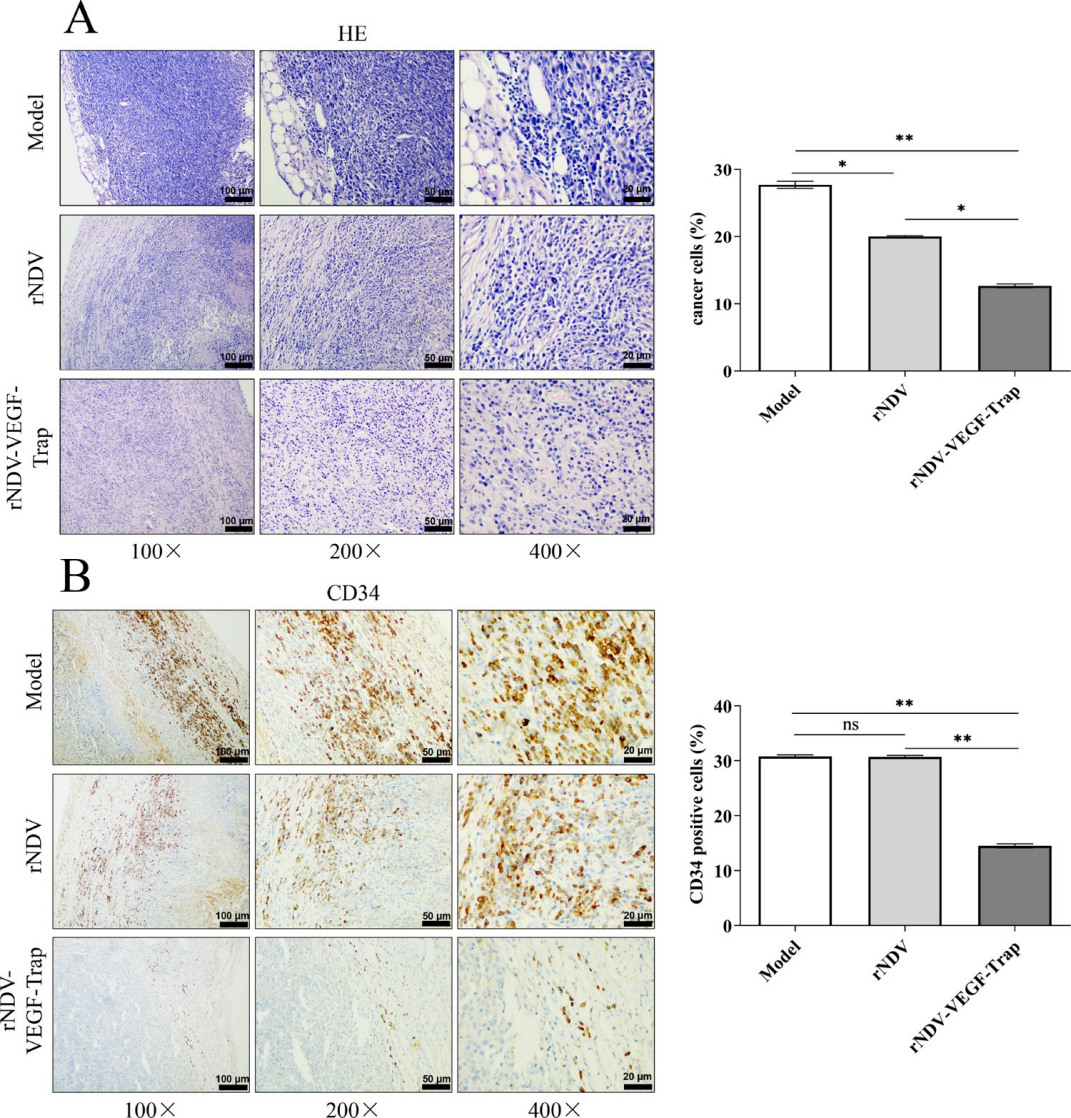
rNDV-VEGF-Trap enhances oncolytic effect in CT26 model. Six-week-old female BALB/c mice were injected with 10^6^ CT26 cells in the right groin. When the tumor size reached about 100 mm^3^, the mice were intratumorally injected with rNDV or rNDV-VEGF-Trap at 10^7^ pfu. The mice were euthanized and necropsy was performed after 15 days of treatment. **(A)** H&E staining of CT26 tumor section after treatment. **(B)** CD34 in the tumor tissues were visualized by IHC assay. The proliferation of vascular endothelial cells were shown in brown. Model: mice treated with allantoic fluid. N = 3. Magnification: 100×, 200×, 400×.

As shown in Fig 4A, H&E staining tumor sections showed that tumors derived from rNDV-VEGF-Trap group demonstrated significant suppression to tumor cell viability than rNDV and model group. Then we evaluated the inhibition of rNDV-VEGF-Trap on vascular endothelial cell proliferation in the tumor tissue by CD34 IHC assay. Compared with model and rNDV group, we found that the expression of CD34 was significantly decreased by treatment with rNDV-VEGF-Trap (Fig 4B). These data suggest that rNDV-VEGF-Trap exhibits an enhanced therapeutic efficacy in colon cancer model.

### rNDV-VEGF-Trap modulates the related protein expression of angiogenesis and apoptosis

**Fig 5:**
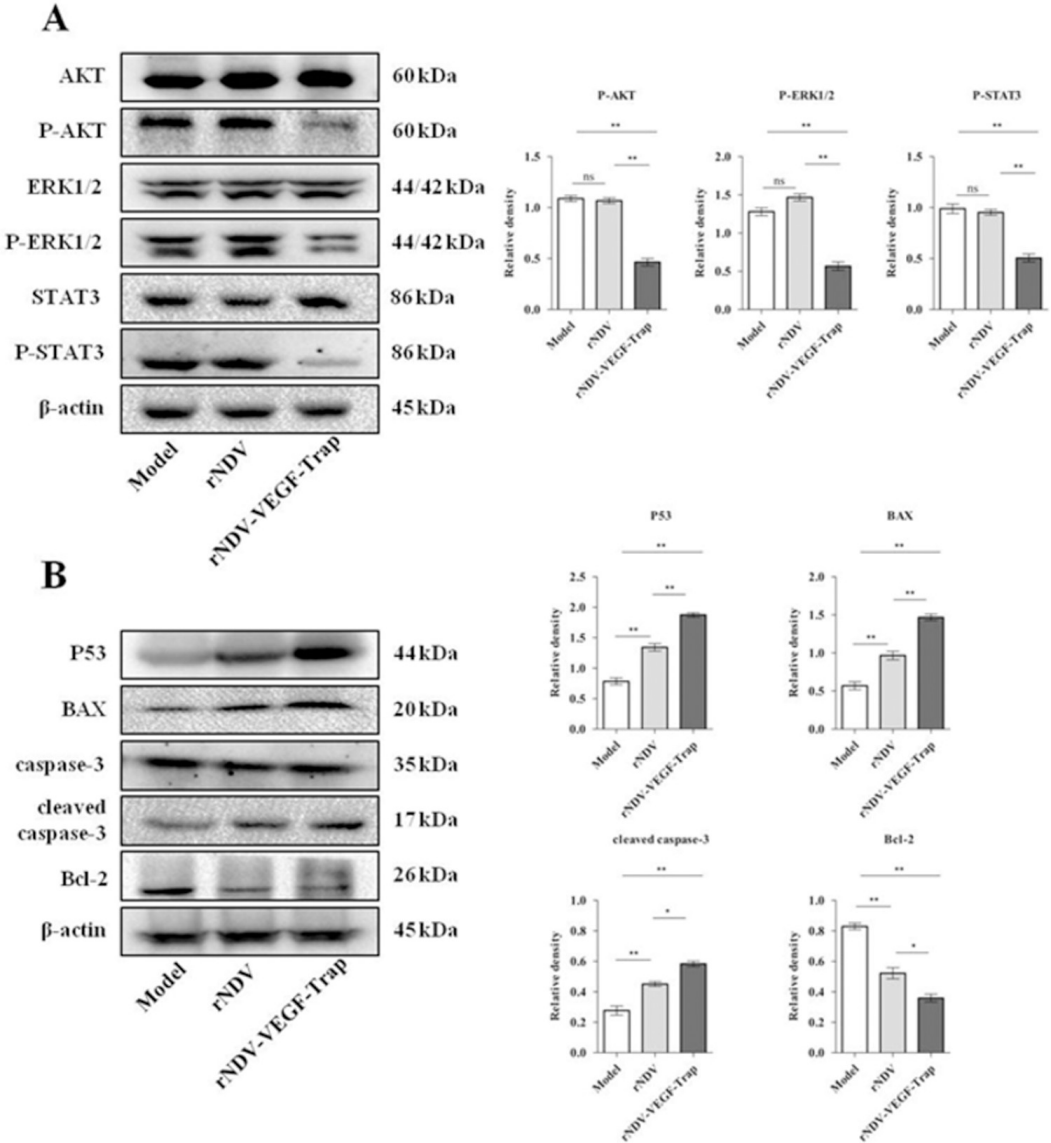
rNDV-VEGF-Trap inhibits the phosphorylation of the VEGF signaling pathway related proteins AKT, ERK1/2, STAT3 and mediates the expression of the apoptosis-related proteins P53, Bcl-2, BAX, caspase-3. Six-week-old female BALB/c mice were injected with 10^6^ CT26 cells in the right groin. When the tumor size reached about 100 mm^3^, the mice were intratumorally treated with rNDV or rNDV-VEGF-Trap at 10^7^ pfu. The mice were euthanized and necropsy was performed after 15 days of treatment. CT26 tumor tissue (each group 0.5 g) was homogenized, the supernatant was collected after centrifugation. The expression of the VEGF signaling pathway related proteins and the apoptosis-related proteins in supernatant were detected by Western blot. **(A)** Treatment with rNDV-VEGF-Trap significantly reduced the expression levels of P-AKT, P-ERK and P-STAT3 compared with rNDV. The relative protein expression levels were expressed as the ratio of P-AKT to AKT, P-ERK1/2 to ERK1/2 and P-STAT3 to STAT3 (arbitrary units). **(B)** rNDV-VEGF-Trap significantly increased the expression levels of P53, BAX and cleaved caspase-3 and significantly downregulated the expression level of Bcl-2 compared with rNDV. β-actin was used as reference to quantify the protein bands by densitometry using ImageJ software. All data were expressed as mean ±SEM, N  =  3, (ns, no significant; *P < 0.05; **P < 0.01) compared to the model.

To elucidate the antiangiogenic mechanisms of rNDV-VEGF-Trap, the related proteins of VEGF signaling pathway were analyzed by Western blot. As shown in Fig 5A, the expression levels of P-AKT, P-ERK and P-STAT in rNDV-VEGF-Trap group were significantly reduced compared with rNDV, while there was no significant difference in the total expression levels of AKT, ERK, and STAT3. The relative protein expression levels were expressed as the ratio of P-AKT to AKT, P-ERK1/2 to ERK1/2 and P-STAT3 to STAT3 (arbitrary units). The results showed that rNDV-VEGF-Trap exhibited the ability to inhibit the related protein activation of VEGF/VEGFR downstream signaling pathways. Besides, the related protein expressions of tumor apoptosis were measured by Western blot. As shown in Fig 5B, the expression levels of P53, BAX and cleaved caspase-3 were significantly increased and the expression level of Bcl-2 was significantly downregulated in rNDV group compared with model group. Moreover, compared with rNDV, rNDV-VEGF-Trap significantly increased the expression levels of P53, BAX, and cleaved caspase-3 and significantly downregulated the expression level of Bcl-2. These data suggest that rNDV-VEGF-Trap further promotes apoptosis and anti-angiogenesis by the related proteins of VEGF signaling pathway, consequently further inhibiting the growth of tumor tissue.

### rNDV-VEGF-Trap demonstrates therapeutic safety

**Fig 6:**
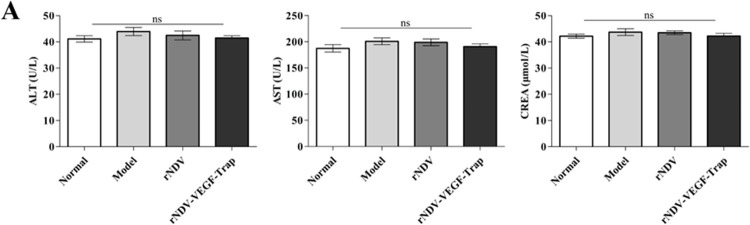
rNDV-VEGF-Trap demonstrates therapeutic safety. At the end of the animal experiment, whole blood was collected through the eyeballs of the experimental mice. Then the serum was isolated from whole blood for determination of serum concentrations of AST, ALT and creatinine **(A)**. The serum biochemistry data of the experimental mice values were expressed as mean ± SEM. ALT: alanine aminotransferase; AST: aspartate transaminase; CREA: creatinine; Normal: normal mice. (ns: no significance).

Previous papers demonstrate that administration of VEGF-Trap exhibits common adverse events in clinical, including diarrhoea, decreased appetite, weight loss, haemorrhage, deteriorated creatinine levels, AST and ALT [41]. In this study, our results showed these common adverse events were not significantly observed in each group (normal, model, rNDV, rNDV-VEGF-Trap) of the CT26-bearing mice (Fig 6 and Table 1). These results suggest that rNDV-VEGF-Trap exhibits therapeutic safety.

**Table 1:** Diarrhoea, appetite, weight and haemorrhage situation of CT26-bearing mice throughout the treatment phase.

	Normal	Model	rNDV	rNDV-VEGF-Trap
Diarrhoea	no	no	no	no
Decreased appetite	no	no	no	no
Weight (g)	26±1.3	25±0.5	24±1.6	25±0.8
Haemorrhage	no	no	no	no

Data was presented from ten mice per group. no: No clinical symptoms were observed during the experimental period.

## Discussion

As one of the oncolytic viruses, Newcastle disease virus represents a new class of therapeutic agents of cancer therapy [42]. But, due to anti-angiogenesis effect of NDV is still limited, the clinical application of NDV isn’t satisfactory [43]. At present, VEGF-Trap, bevacizumab, cetuximab and panitumumab show promising in antiangiogenic therapy, in which VEGF-Trap has the advantages of significant therapeutic effect and long Half-Life compared with others [44, 45]. However, VEGF-Trap inevitably arises multiple adverse effects in the clinical treatment of colon cancer [37]. Previous study shows that researchers use recombinant AAV2 as a delivery vehicle to achieve long-lasting expression of VEGF-Trap protein in a mouse model, simultaneously suppressing primary tumor growth and preventing the metastases of tumors [46]. However, the therapeutic effect need to be optimized for improving the survival rate. Therefore, these problems indicate that it is necessary to find a potential candidate for the treatment of colon cancer.

In this study, we generated rNDV-VEGF-Trap using reverse genetics. As anticipated, the kinetics of replication of rNDV-VEGF-Trap did not show significant changes and rNDV-VEGF-Trap successfully expressed VEGF-Trap protein in the CT26 tumor cells and the tumor tissues. NDV specifically replicates in cancer cells rather than in normal cells because of the defective IFN signal pathways in cancer cells [24]. EA. hy926 cells are hybridized by HUVEC cells and lung cancer cells A549, which have the dual characteristics of vascular endothelial cells and lung cancer cells [47]. Therefore, we choose EA. hy926 cells as antiangiogenic model. MTT results showed that the EA.hy926 cells were infected with the virus at 10 MOI for 48h, the inhibitory rate treated by rNDV-VEGF-Trap was 85.37% and rNDV was 74.26%. The results showed that rNDV-VEGF-Trap enhanced inhibitory efficacy of the virus. Results of scratch test show that the EA.hy926 cells were infected with the virus at 1 MOI for 48h, the relative mobility rate treated by rNDV-VEGF-Trap was 12.1% and rNDV was 26.5%. The results showed that rNDV-VEGF-Trap enhanced the ability of the virus to inhibit the migration of EA.hy926 cells. The results are similar to the previously report that VEGF-Trap protein inhibits vascular endothelial cell proliferation and migration [36]. In summary, rNDV-VEGF-trap was successfully generated in this study. *In vitro*, rNDV-VEGF-Trap further inhibits the proliferation and migration of vascular endothelial cells.

Based on the encouraging *in vitro* results, we investigated the therapeutic efficacy of rNDV-VEGF-Trap in CT26-bearing mice. The results showed that treatment with rNDV-VEGF-Trap reduced tumor volume of CT26-bearing mice by more than 3 folds and tumor weight by more than 4 folds. The results indicate that rNDV-VEGF-Trap effectively inhibits tumor growth. Previous studies have shown that tumor tissue angiogenesis is caused by excessive and abnormal proliferation of vascular endothelial cells [48, 49]. As surface markers of vascular endothelial cells, CD34 is commonly applied to detect the situation of vascular proliferation [50, 51]. Immunohistochemistry results of CD34 showed rNDV-VEGF-Trap significantly reduced the number of vascular endothelial cells in the tumor tissues of the tumor-bearing mice. The results indicate that rNDV-VEGF-Trap has a significant antiangiogenic ability in tumor tissue than rNDV. In summary, rNDV-VEGF-Trap significantly improves the inhibiting effect on the tumor growth of the CT26 tumor-bearing mice.

Based on the results of *in vivo* experiments, we have shown that rNDV-VEGF-Trap has the ability to inhibit tumor growth and angiogenesis in the tumor. However, the mechanisms of rNDV-VEGF-Trap inhibiting angiogenesis and tumor growth are not clear. Previous studies have shown that VEGF-Trap binds VEGF and prevents its binding and activation of VEGF receptor [36]. As a pivotal and pervasive pro-angiogenic factor, VEGF regulates multiple aspects of tumor angiogenesis through VEGF receptor [52]. In response to its binding, VEGFR initiates signaling through the Raf-MEK-ERK, PI3K-AKT kinase cascades [53–55] and STAT3 [56]. Signaling through these pathways promotes proliferation, survival and chemotaxis in endothelial cells and ultimately produces the characteristic effects of VEGF on vessels, such as increased vascular permeability and angiogenesis [57, 58]. In general, these signaling pathways in endothelial cells are not activated in normal mouse organs, but only in blood vessels of tumor tissues. In addition, these signaling pathways are activated not only by VEGF-VEGF receptor, but also by other angiogenic promoters (such as basic FGF) and thus, may serve as general indicators of angiogenic activation [59, 60]. To determine whether these signaling pathways are blocked in tumor vessels *in vivo* by rNDV-VEGF-Trap, we employed Western blot for detection of ERK (P-ERK), AKT (P-AKT) and STAT3 (P-STAT3) in the tumor tissue of mice. The results showed that treatment with rNDV-VEGF-Trap significantly reduced the ratios of P-AKT/AKT, P-ERK/ERK and P-STAT3/STAT3. This suggests that rNDV-VEGF-Trap inhibits tumor tissue angiogenesis by blocking the activation of VEGF-VEGF receptor related signaling pathways. Previous studies demonstrated that VEGF-Trap indirectly induces apoptosis of tumor cells by inhibiting angiogenesis [36]. In order to demonstrate whether rNDV-VEGF-Trap enhances virus-mediated tumor tissue apoptosis, we detected the expression levels of P53, BAX, Bcl-2 and cleaved caspase-3 in tumor tissues by Western blot. The results showed that the expression levels of P53, BAX, and cleaved caspase-3 in rNDV-VEGF-Trap treatment group were significantly increased and the expression levels of Bcl-2 were significantly decreased compared with rNDV. This indicates that rNDV-VEGF-Trap further promotes the apoptosis of tumor tissue. In this study, we preliminarily confirmed the mechanism by which rNDV-VEGF-Trap inhibits angiogenesis and tumor growth. First, rNDV-VEGF-Trap inhibits the activation of AKT, ERK and STAT3 signaling pathways by blocking VEGF and VEGF receptor binding, leading to a reduced tumor angiogenesis. Secondly, the VEGF-Trap effectively enhanced the viral-vector-mediated tumor tissue apoptosis by inducing the expression levels of apoptotic related protein P53, BAX, Bcl-2 and cleaved caspase-3. The other potential mechanisms will be further investigated in next experiment.

In previous studies, purified VEGF-Trap proteins were applied to cancer therapy, and the main problem was the side effects [37]. In order to evaluate the adverse reactions of rNDV-VEGF-Trap, toxicity analysis was performed. Our results showed that there were no obvious clinical symptoms between infected and uninfected groups, including diarrhoea, appetite, weight and haemorrhage. Besides, analysis of serum chemistries showed no significant changes in AST, ALT and creatinine levels. Finally, we observed all key organs and didn’t find obvious lesions. The virus residues were not detected in the normal tissues at the end of experiment (S1 Fig). At the same time, some published papers have demonstrated NDV safety [24, 38]. The purpose of this study was to evaluate the enhanced therapeutic effect of the recombinant virus expressing VEGF-Trap on colon cancer. Furthermore, additional experiments will be performed, for example: improving injected frequency and survival rates study. These data suggest that administration of rNDV-VEGF-Trap is relatively safe in the treatment of colon cancer.

In conclusion, we show a promising oncolytic agent rNDV-VEGF-Trap consisting of an ideal vector and a suppressor gene VEGF-Trap. Our results demonstrate that rNDV-VEGF-Trap exhibits a better anticancer efficacy than rNDV in CT26 model. In addition, rNDV-VEGF-Trap effectively reduces the adverse reactions induced by the treatment of purified VEGF-Trap and demonstrates safety. Thereby, this study provides a potential candidate for the treatment of colon cancer.

## Supporting information

S1 Fig rNDV-VEGF-Trap residue were not detected in normal tissues at the end point. At the end of experiment, we performed the hemagglutination test (HA). The results showed that the HA titer in the tumor tissue was 1:32 and in the normal tissues was none.
(DOCX)

S1 Raw images (PDF)
