# Supplementary material for: Newcastle disease virus expressing an angiogenic inhibitor exerts an enhanced therapeutic efficacy in colon cancer model
Source: PLoS One. 2022 Apr 5;17(4):e0264896. doi: 10.1371/journal.pone.0264896 (PMC8982889; doi:10.1371/journal.pone.0264896)

**S1 Fig. rNDV-VEGF-Trap residue were not detected in normal tissues at the end point**. At the end of experiment, we performed the hemagglutination test (HA). The results showed that the HA titer in the tumor tissue was 1:32 and in the normal tissues was none.


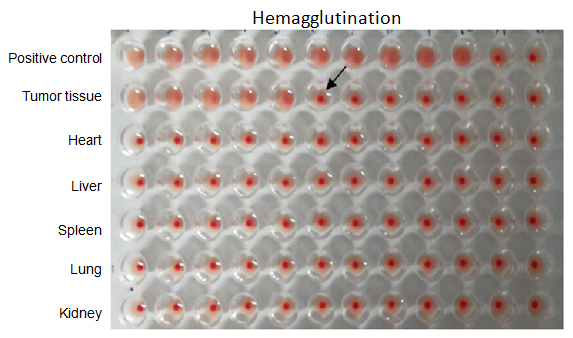

Supplement: S1 Fig — At the end of experiment, we performed the hemagglutination test (HA). The results showed that the HA titer in the tumor tissue was 1:32 and in the normal tissues was none. (DOCX) [file pone.0264896.s001.docx]
