# Supplementary material for: Newcastle disease virus expressing an angiogenic inhibitor exerts an enhanced therapeutic efficacy in colon cancer model
Source: PLoS One. 2022 Apr 5;17(4):e0264896. doi: 10.1371/journal.pone.0264896 (PMC8982889; doi:10.1371/journal.pone.0264896)
Supplement: S1 Raw images — (PDF) [file pone.0264896.s002.pdf]

A. The expression of VEGF-Trap proteins in CT26 cells

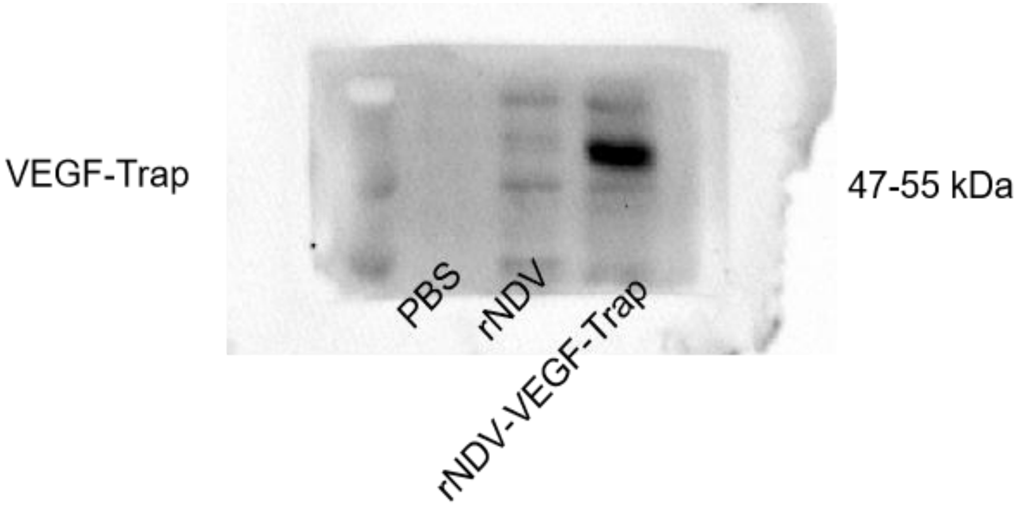

Panel **A** represents Western blot analysis shown in **Fig1C**. Every lane in every protein image was a same group of cells. The first lane was PBS control. The second lane was rNDV treatment. The third lane was rNDV-VEGF-Trap treatment.The data of manuscript were these original data.

B. Expression of VEGF-Trap protein *in vivo*.

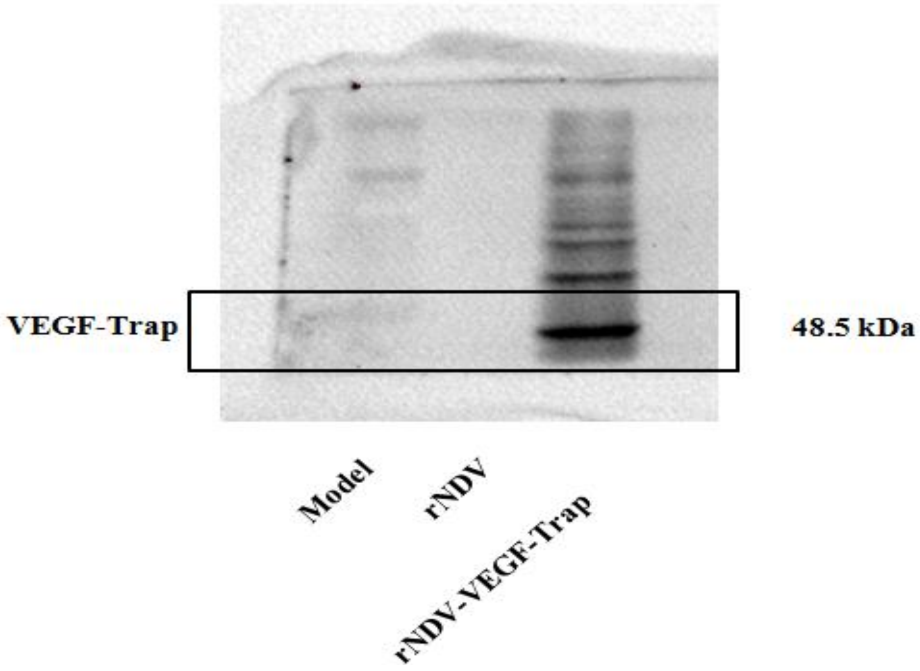

Panel **B** represents Western blot analysis shown in **Fig1D**. Every lane in every protein image was a sample of mice. The first lane was model group. The second lane was rNDV treatment group. The third lane was rNDV-VEGF-Trap treatment group.The data of manuscript were these original data.

C. The phosphorylation of the VEGF signaling pathway related proteins AKT, ERK1/2, STAT3

AKT

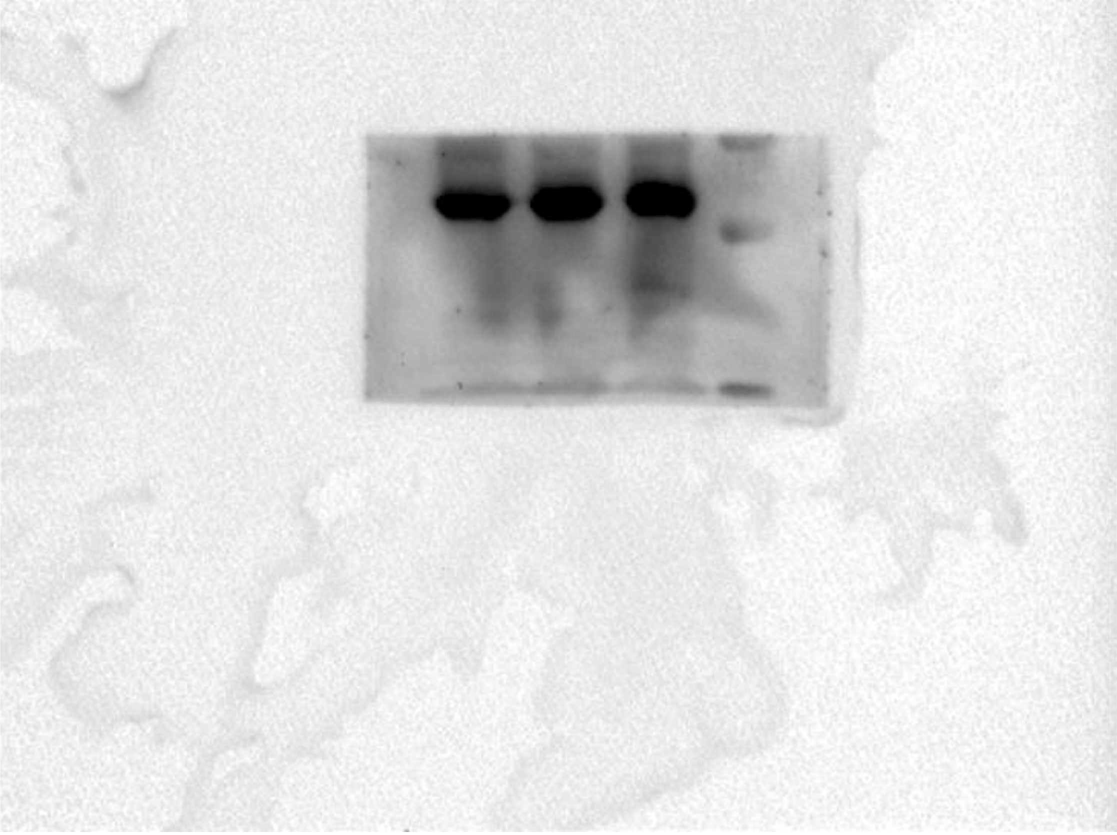

P-AKT

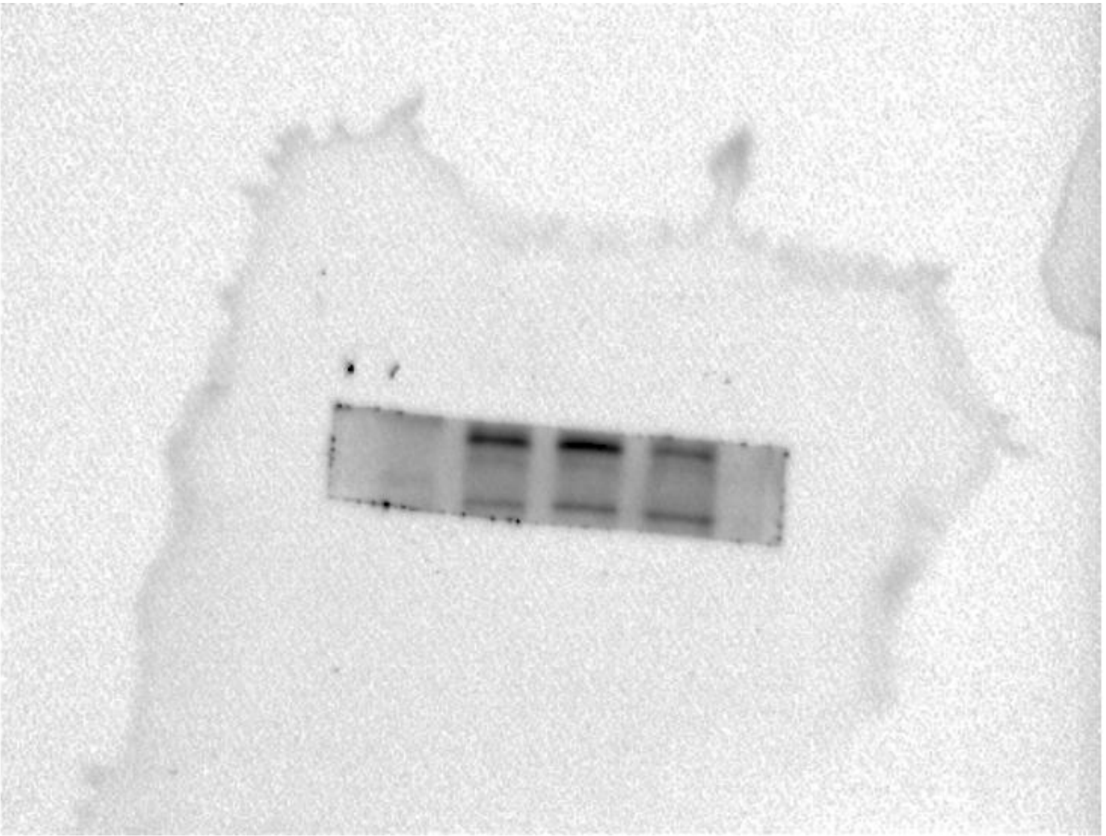

ERK1/2

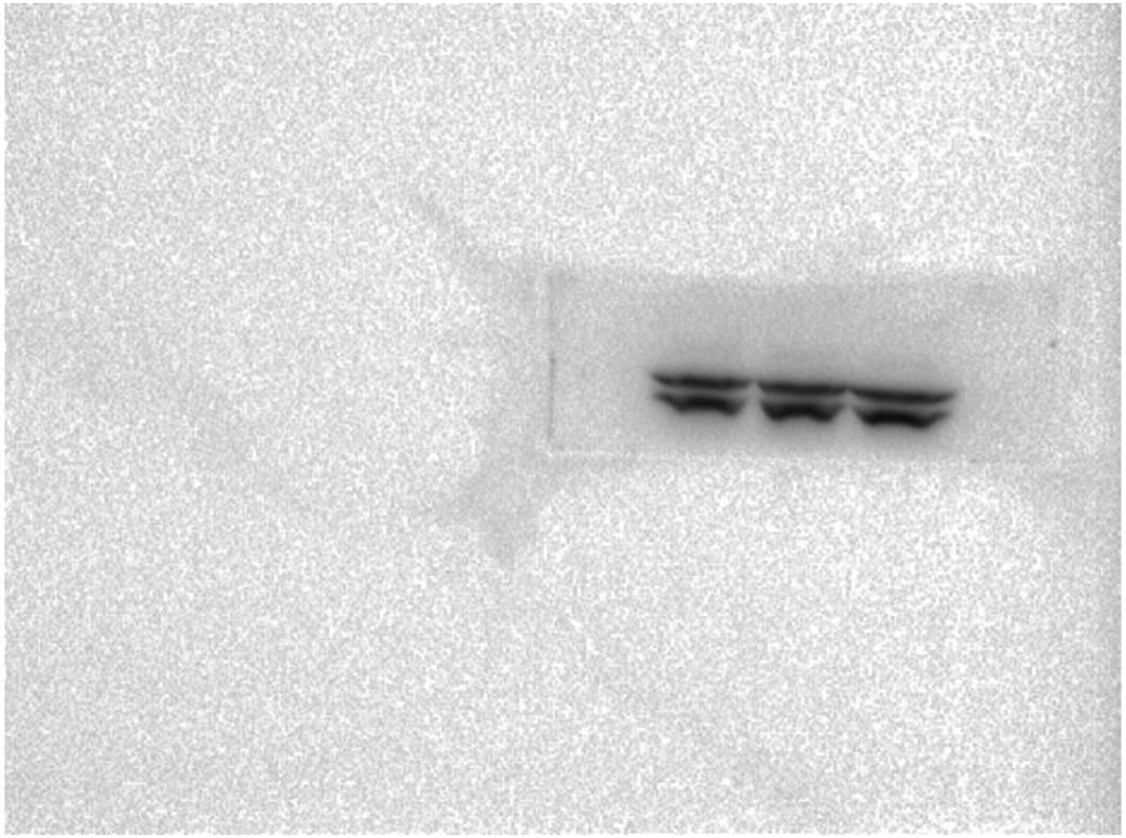

P-ERK1/2

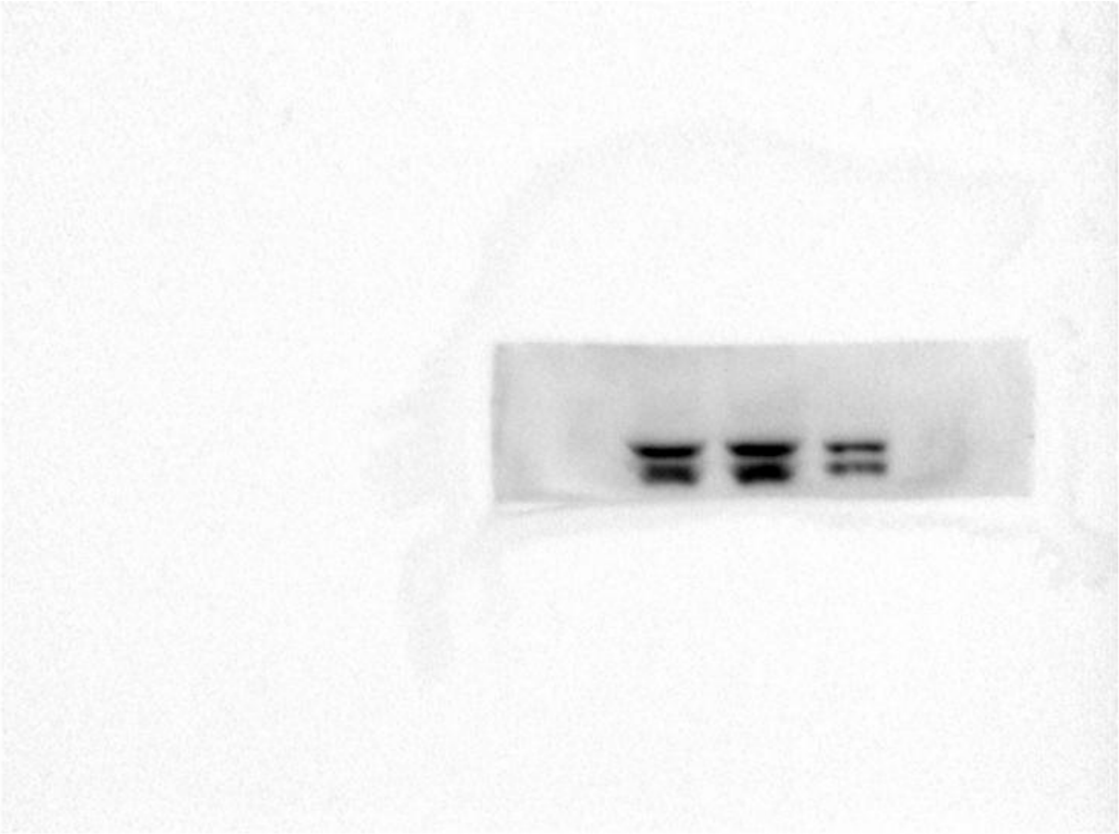

STAT3

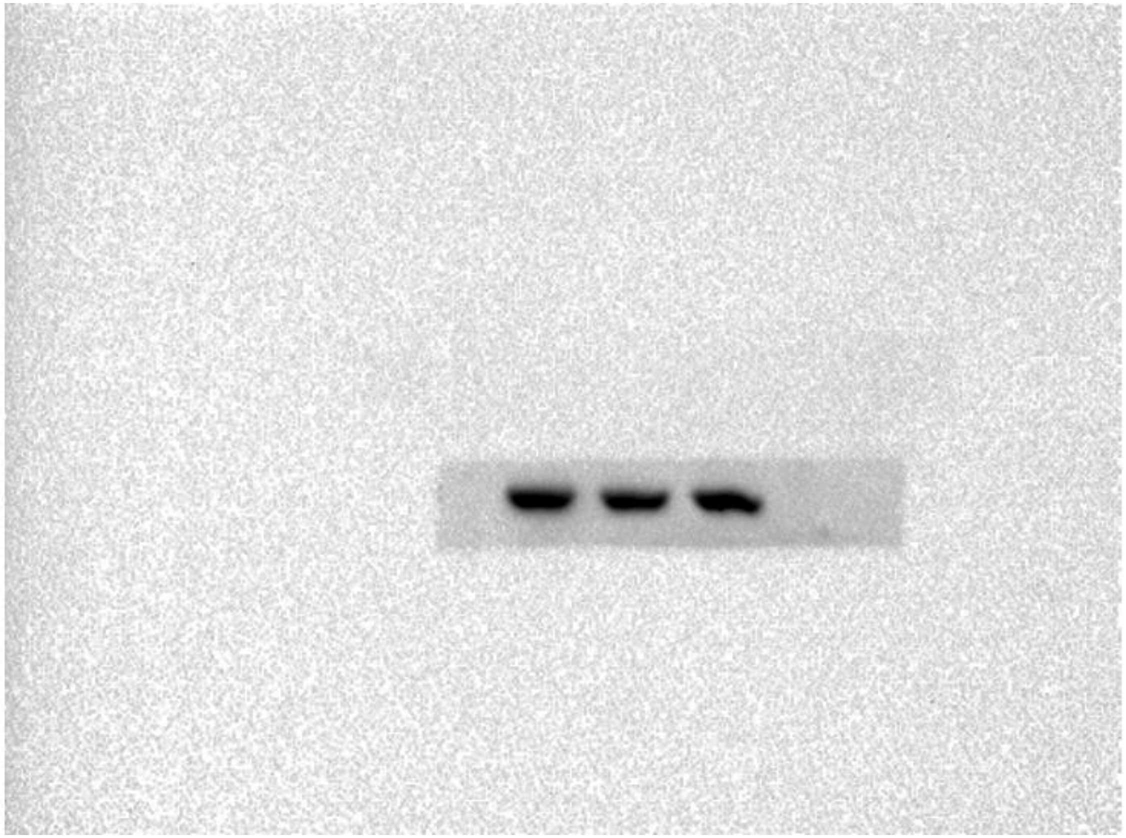

P-STAT3

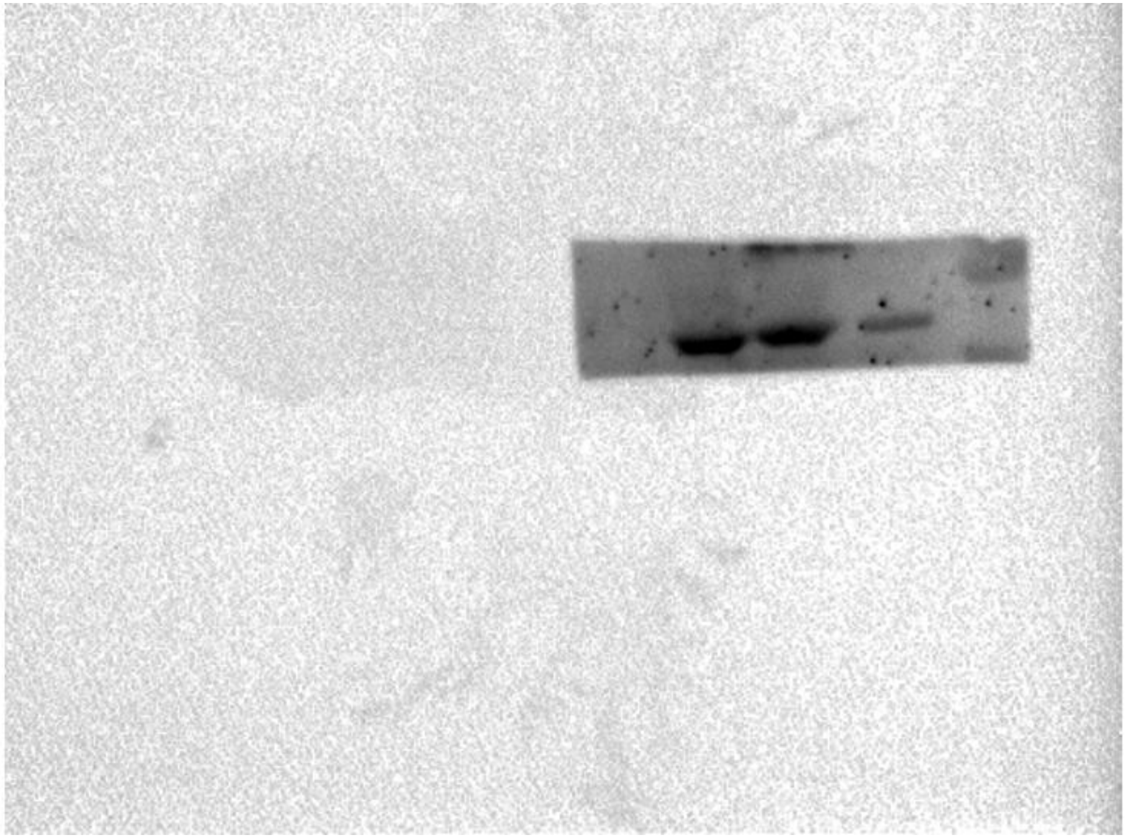

β-actin

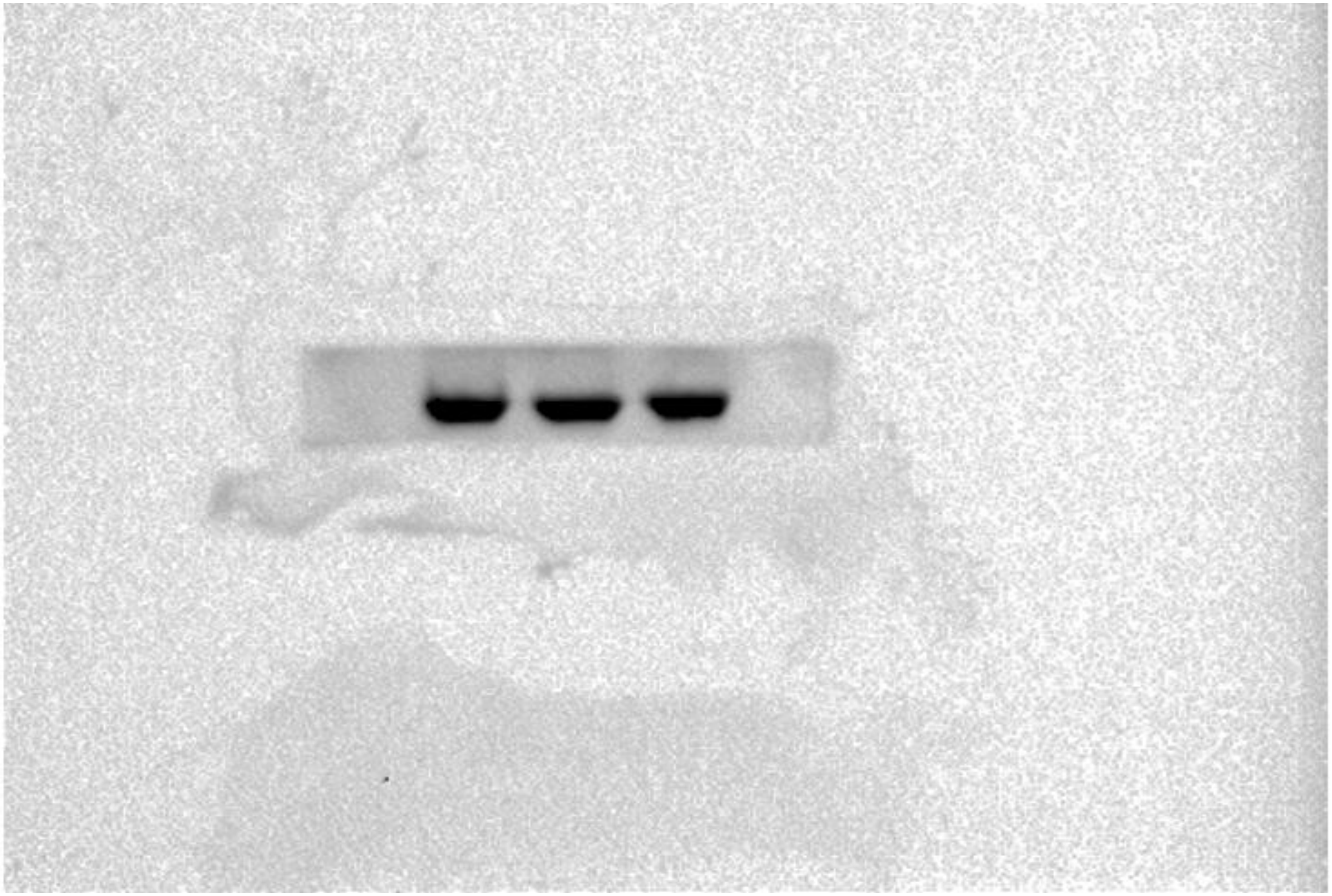

Panel **C** represents Western blot analysis shown in **Fig. 5A**. Every lane in every protein image was a same group of the tumor tissue mice. The first lane was allantoic fluid treatment. The second lane was rNDV treatment. The third lane was rNDV-VEGF-Trap treatment.The data of manuscript were these original data.

D. The expression of the apoptosis-related proteins P53, BAX, Bcl-2, caspase-3

P53

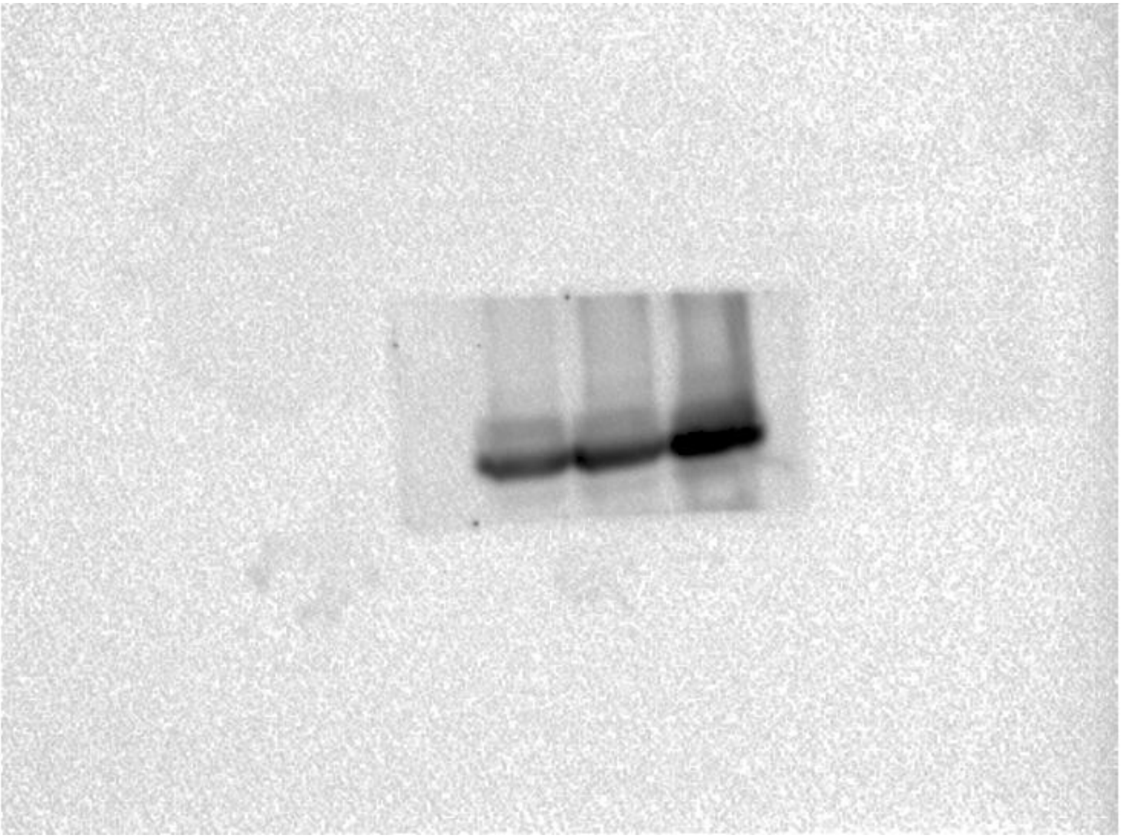

BAX

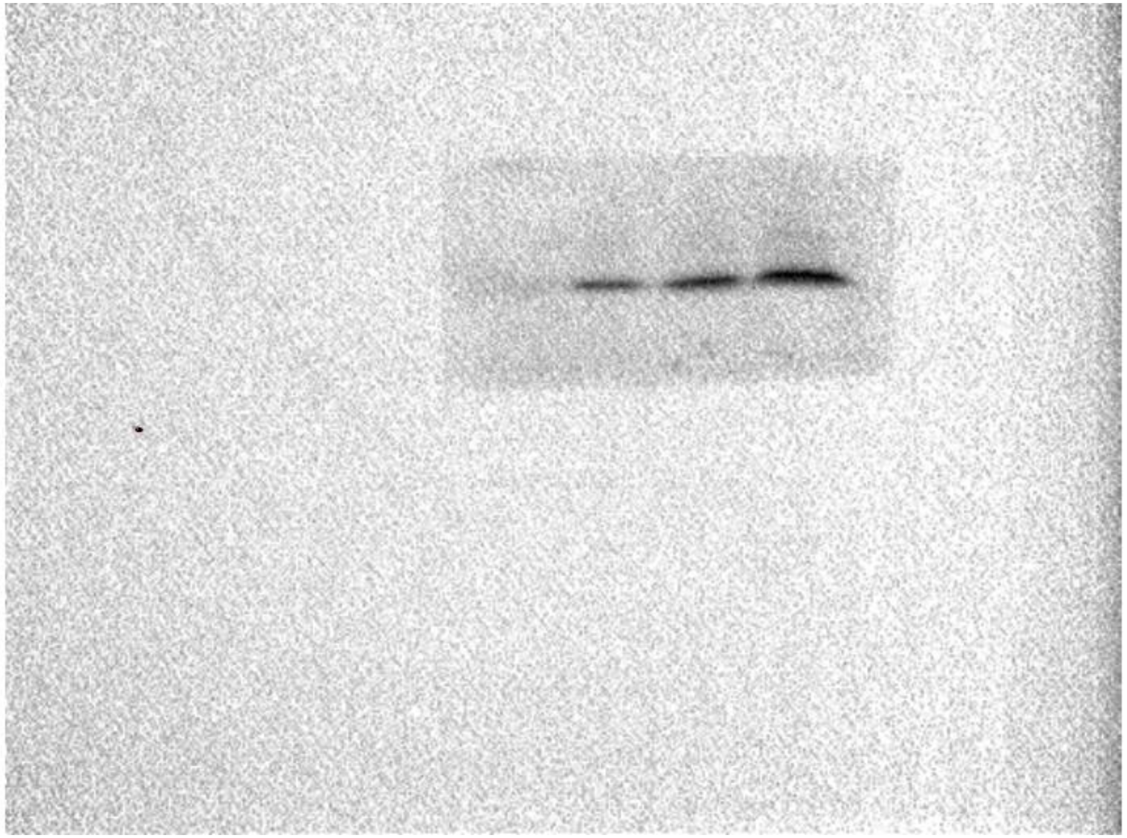

caspase-3

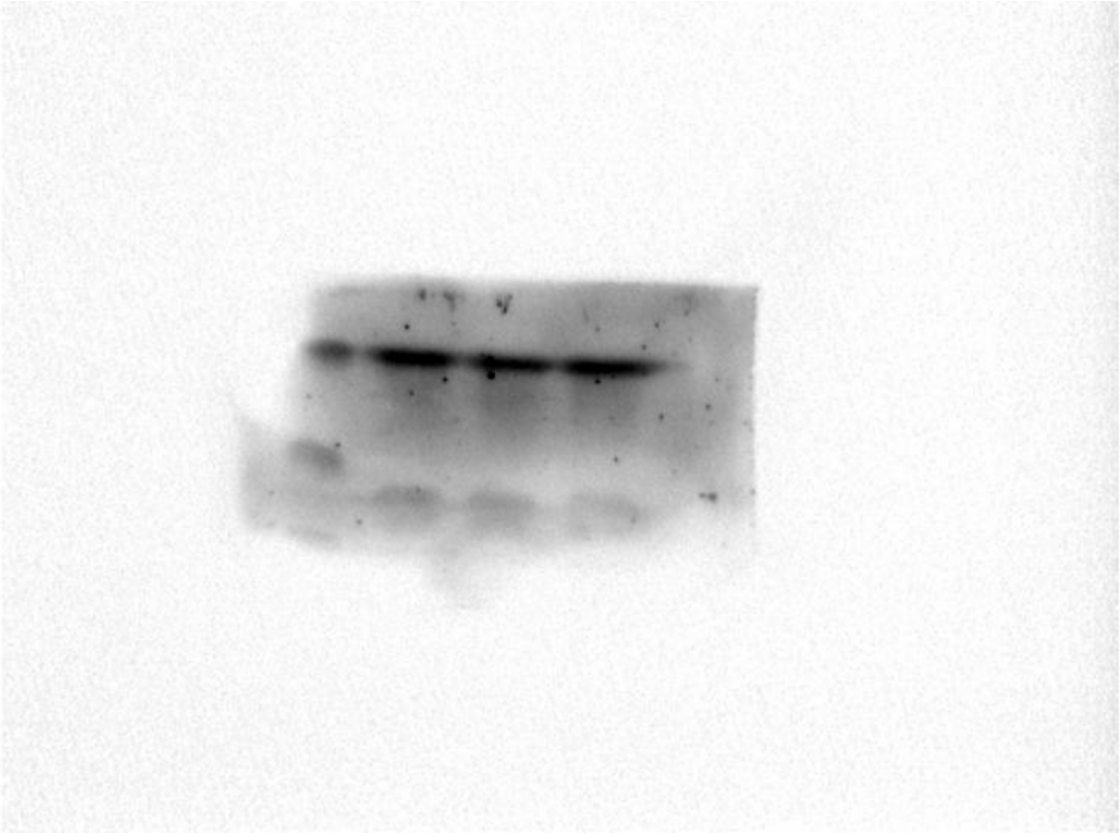

cleaved caspase-3

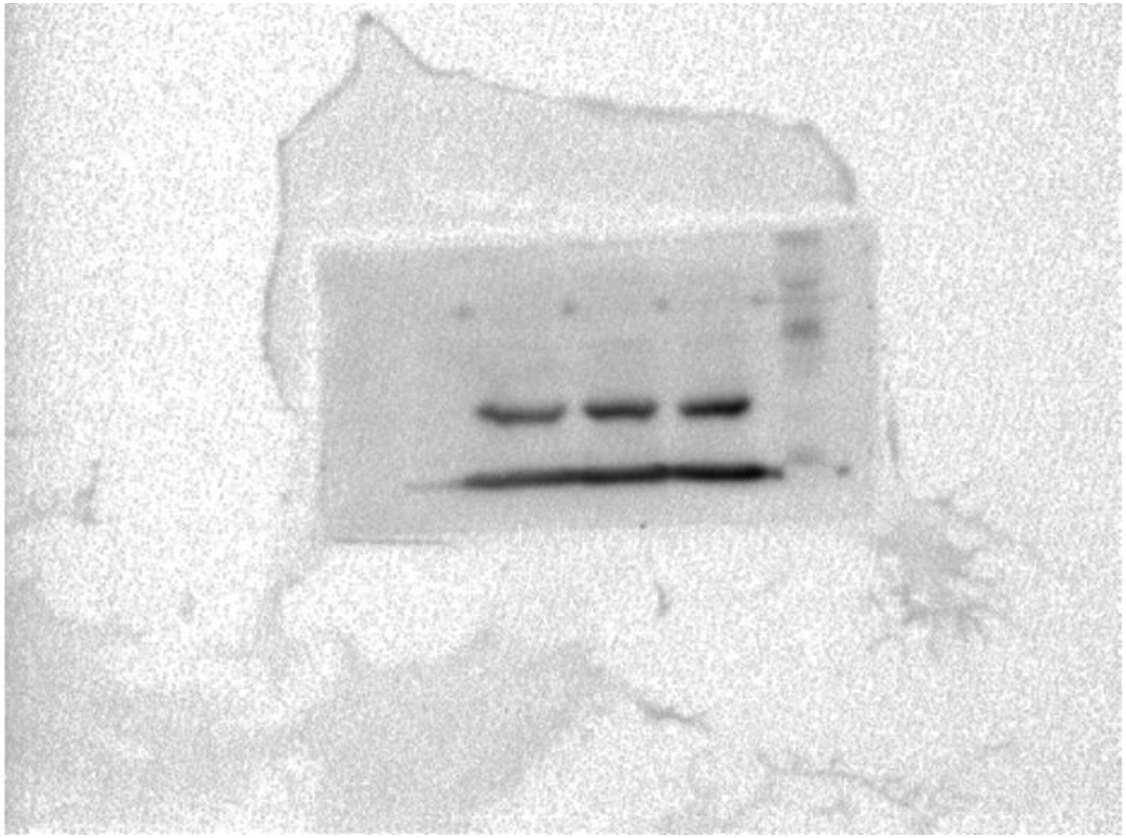

Bcl-2

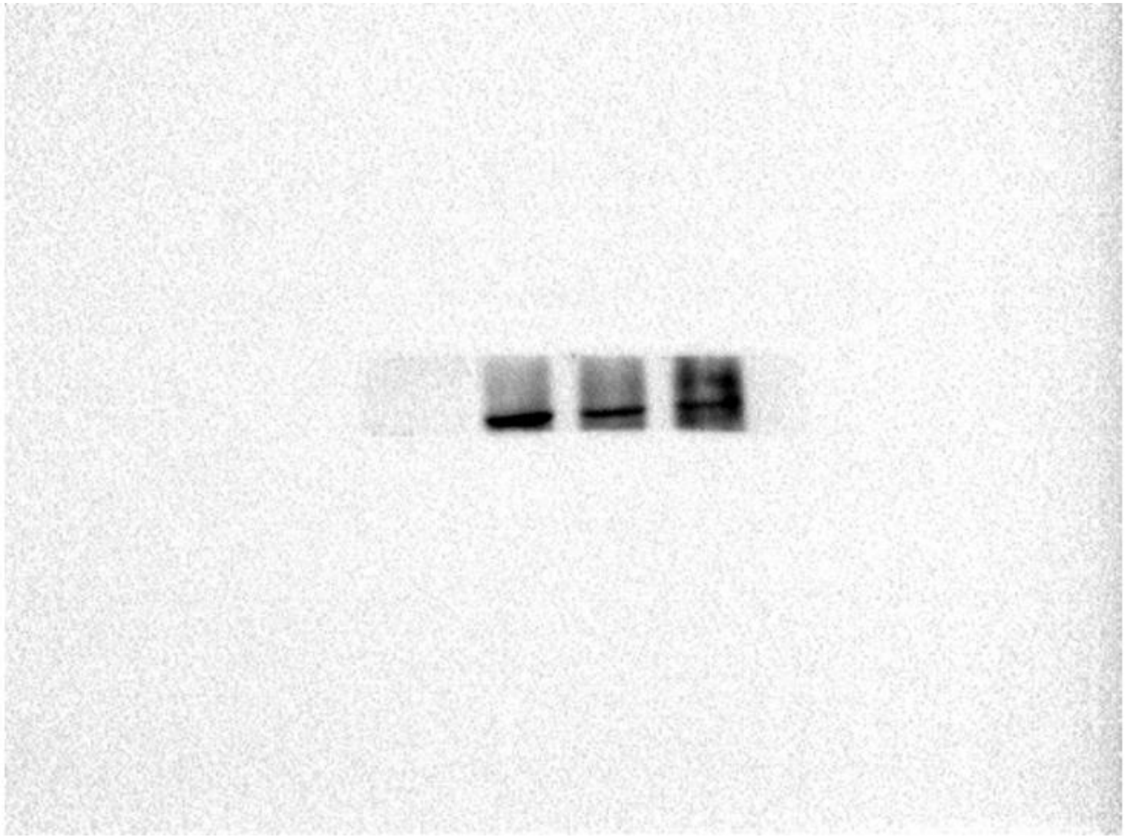

β-actin

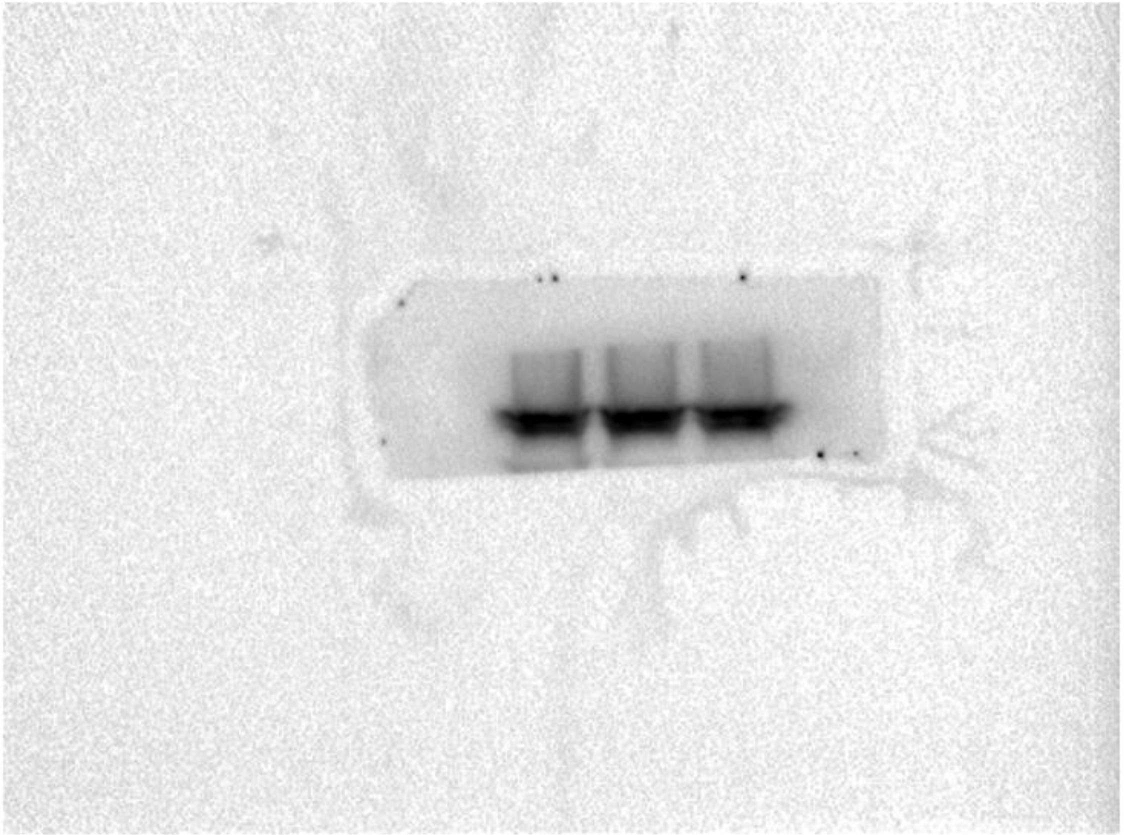

Panel **D** represents Western blot analysis shown in **Fig. 5B**. Every lane in every protein image was a same group of the tumor tissue mice. The first lane was allantoic fluid treatment. The second lane was rNDV treatment. The third lane was rNDV-VEGF-Trap treatment.The data of manuscript were these original data.
